# From lung to breast: a rare case of metastatic lung adenocarcinoma presenting as a breast lump in a male patient

**DOI:** 10.1093/jscr/rjaf148

**Published:** 2025-03-25

**Authors:** Elias Edward Lahham, Huda Maher Masri, Zeina Adnan Farhoud, Abrar Nidal Neiroukh, Mahmoud Ramahi, Farah Awad, Marwan Qubaja

**Affiliations:** Department of Radiation Oncology, Augusta Victoria Hospital, Martin Buber Jerusalem 9119101, East Jerusalem, Palestinian Authority, Palestine; Faculty of Medicine, Al-Quds University, Main Campus, Abu Dis, PO Box 89, Palestine; Faculty of Medicine, Al-Quds University, Main Campus, Abu Dis, PO Box 89, Palestine; Faculty of Medicine, Al-Quds University, Main Campus, Abu Dis, PO Box 89, Palestine; Department of Radiology, Augusta Victoria Hospital, Martin Buber Jerusalem 9119101, East Jerusalem, Palestinian Authority, Palestine; Department of Oncology, Augusta Victoria Hospital, Martin Buber Jerusalem 9119101, East Jerusalem, Palestinian Authority, Palestine; Department of Pathology, Augusta Victoria Hospital, Martin Buber Jerusalem 9119101, East Jerusalem, Palestinian Authority, Palestine

**Keywords:** metastatic lung adenocarcinoma, breast lump, breast metastasis

## Abstract

Pulmonary adenocarcinoma, the most common subtype of non-small cell lung cancer, frequently metastasizes to the brain, liver, bones, and adrenal glands. However, breast metastases are exceedingly rare, accounting for 0.2%–1.3% of extramammary metastases, with only 0.1% of secondary breast malignancies originating from the lung. This case report presents a 56-year-old non-smoking male who presented with a unilateral retro-areolar breast lump. Further evaluation revealed ipsilateral axillary lymphadenopathy, and diagnostic biopsy and immunohistochemistry confirmed the lung origin of the breast lesion. This study emphasizes the significance of taking into account breast metastasis in the differential diagnosis of breast lesions in male patients, particularly those with a known primary malignancy. It highlights the need to recognize breast lumps as a potential presentation of secondary breast malignancy.

## Introduction

Pulmonary cancer is the leading cause of cancer-related death worldwide with a mortality rate of 21% in both genders [1]. The majority of lung cancers are non-small cell lung cancer (NSCLC), accounting for 84% of cases, whereas 13% of lung cancers are small cell lung cancer (SCLC) [2].

The most prevalent type of primary lung cancer is adenocarcinoma which is categorized as NSCLC. It constitutes 40% of lung cancers and is the main subtype to be diagnosed in nonsmokers. As with any type of lung cancer, the most common predisposing factor for lung adenocarcinoma is cigarette smoking, Moreover, a history of lung cancer in the family members and occupational lung exposures carry a significant risk for it [3].

Frequent sites of lung adenocarcinoma metastasis are liver, adrenal glands, bone and brain [4]. It rarely metastasizes to the breast tissue. The incidence of breast metastasis from an extra mammary cancer -including lung cancer- ranges from 0.2% to 1.3% [5] with the most common primary cancers being malignant melanoma, bronchial lung cancer, gynecological cancers, and gastrointestinal tumors [1]. This condition is observed six times more common among females, however, it’s extremely uncommon among males [6].

## Clinical presentation

**Figure 1:**
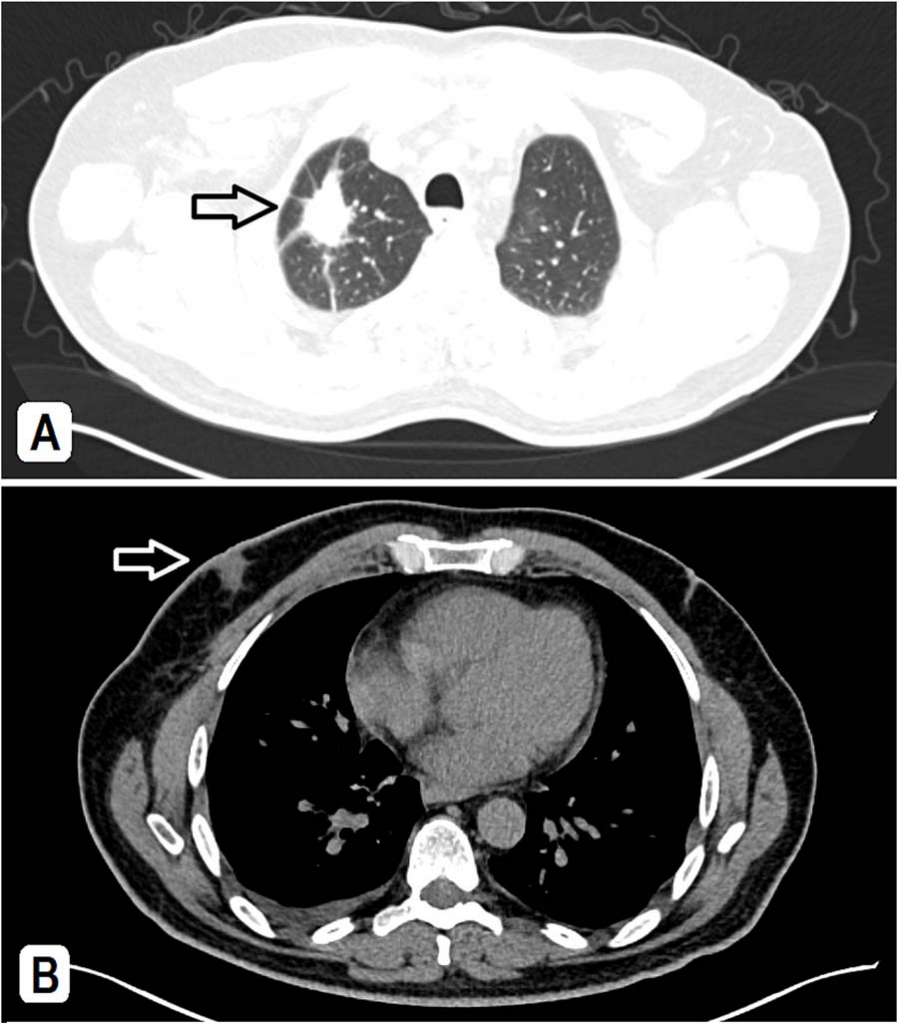
(A) A CT scan showed a spiculated lesion in the right lung apex with adjacent atelectatic bands, pathologically enlarged hilar, mediastinal, and right-sided axillary lymph nodes, the largest measuring 1.5 cm. (B) The mediastinal window of CT at the chest level shows an asymmetry of breast tissue with right-sided retro-areolar soft tissue density.

**Figure 2:**
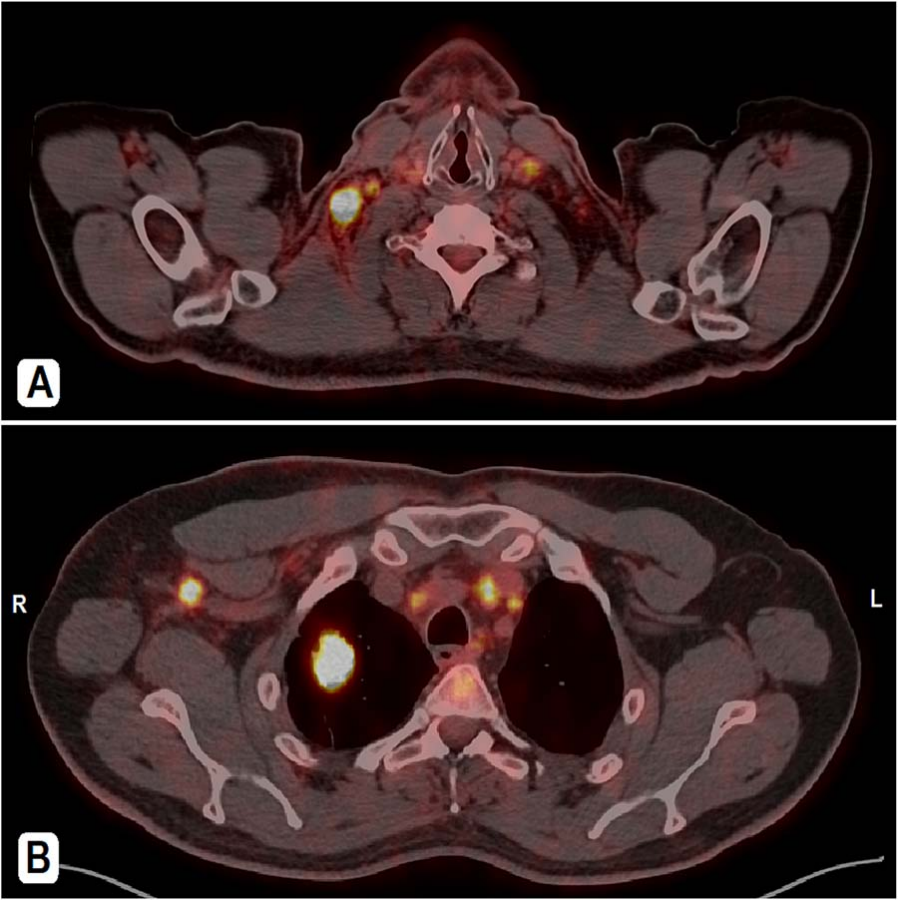
PET-CT scan. (A) At the neck level showing intensely hypermetabolic enlarged cervical lymph nodes. (B) An intensely hypermetabolic right upper lobe lung mass, with metastatic right axillary and mediastinal lymph nodes.

A 56-year-old male patient, married with four offspring, non-smoker, with free past medical and surgical history, presented to his family doctor complaining of a self-palpable, painless lump in the right submandibular area for 1-month duration. Family history included a brother with colon cancer. On examination, a right submandibular lump was noted, measuring ~2 cm, there is also a single painless, retro-areolar palpable breast lump with multiple right palpable lymph nodes. There were no skin or nipple changes. Neck ultrasound revealed right submandibular lymph node enlargement with bilateral prominent suspicious cervical lymph nodes. A CT scan showed a spiculated lesion in the right lung apex with adjacent atelectatic bands, pathologically enlarged hilar, mediastinal, and right-sided axillary lymph nodes. Additionally, there is noted asymmetry of breast tissue with right-sided retro-areolar soft tissue density (Fig. 1A and B). A positron emission tomography (PET) scan emphasized hypermetabolic, mildly prominent, multilevel cervical lymph nodes with intense fluorodeoxyglucose (FDG) uptake (SUVmax 13.3), extending to bilateral supra and right infraclavicular fossae, with the largest node in the right level 1 measuring 1.5 × 1.3 cm. A hypermetabolic right pulmonary apical soft tissue lesion was noted, measuring 2.9 × 2.4 cm with adjacent atelectatic bands, associated with hypermetabolic mediastinal and right axillary lymph nodes with intense FDG uptake (SUVmax 7.2) (Fig. 2A and B).

**Figure 3:**
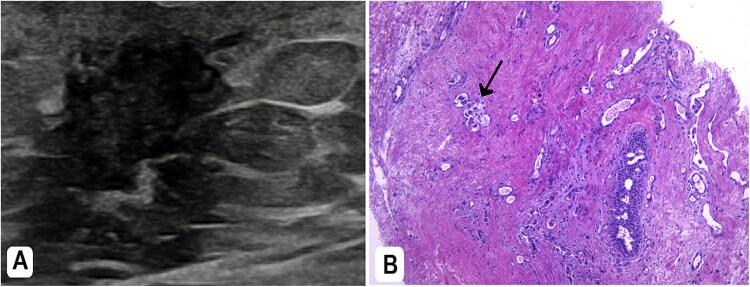
(A) Breast ultrasound showed a right-sided retro areolar irregular antiparallel infiltrative hypo-echogenicity. A biopsy was taken from it. (B) H&E: The breast tissue is infiltrated by large neoplastic cells; adopting glandular pattern in some places (arrow).

**Figure 4:**
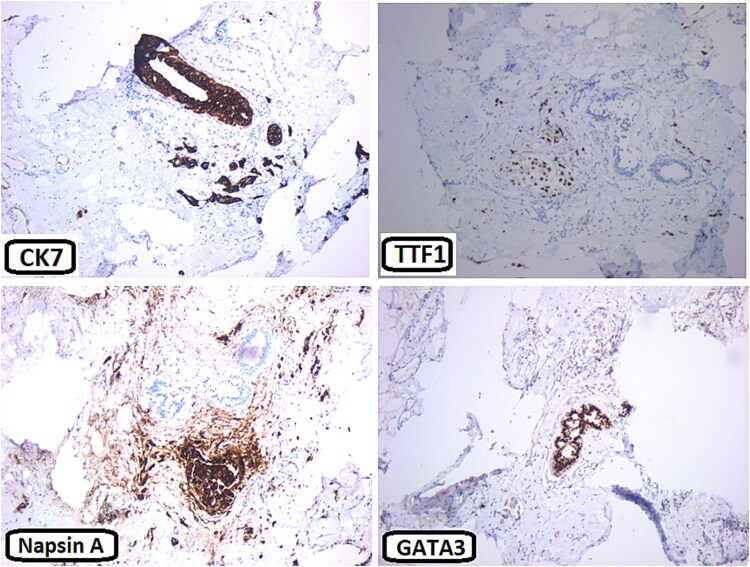
Immunohisochemical stains: The neoplastic cells are positive for CK7, TTF1, and Napsin A. They are negative for GATA3.

The patient underwent a right cervical lymph node tru-cut biopsy, which showed poorly differentiated neoplasm consistent with lung origin. Histopathology showed immunohistochemical positivity for cytokeratin 7 (CK7) and thyroid transcription factor-1 (TTF-1). Molecular analysis revealed ALK-negative, ROS positive, epidermal growth factor receptor (EGFR) mutant (Exon 21 L858R point mutation), and positive PD-L1 30%, all supporting a lung adenocarcinoma diagnosis. The patient was referred to our hospital for further oncology management. Subsequently, a breast ultrasound showed a lump with dendritic and hypoechoic changes, measuring ~1.4 × 1.1 × 1.3 cm. A tru-cut biopsy from the breast mass showed poorly differentiated adenocarcinoma (Fig. 3A and B). Immunohistochemistry (IHC) was positive for CK7, TTF1, Napsin A, PD-L1, ROS1, and negative for GATA3, ER, PR, and Her-2 (Fig. 4).

Right axillary lymph node biopsy and histopathological analysis confirmed metastatic adenocarcinoma, morphologically consistent with lung primary, similar to the breast lesion. As the patient started to complain of pain and increased swelling in the right cervical and submandibular lymph nodes, palliative radiotherapy was administered (a total of 20 Gy in 5 fractions). The patient started on Carboplatin + Pemetrexed (Alimta), a systemic chemotherapy, which was eventually discontinued due to severe neutropenia. As the molecular analysis of the tumor cells showed mutant EGFR, the patient was given Tagrisso (osimertinib), a targeted therapy for EGFR mutation. The overall picture is suggestive of a great response to the treatment.

## Discussion

Metastatic breast involvement is rare, accounting for 0.2%–1.3% of the cases. The majority of mammary gland metastases occur from the contralateral breast [5], and to a lesser extent from extramammary sources including melanoma (29.8%), bronchogenic carcinoma (16.4%), gynecological malignancies (12.7%), and gastrointestinal tumors (9.9%) [1]. NSCLC, particularly adenocarcinoma subtype, is more frequently associated with breast metastasis compared to SCLC [7]. Typically, lung cancer spreads more commonly to organs such as the liver (37%), brain (33%), bones (21%), and adrenal gland (17%) [4]. Breast involvement is exceptionally uncommon in males, forming <1% of all primary and secondary breast cases, and 0.2% of all male cancers, with ~ <1 per 100 000 cases/year, and an increasing rate of 1.1% annually [8]. There are no well-established risk factors linked to the occurrence of breast metastases. The exact role of hormones is controversial, a significant rate of breast metastases has been documented in adolescent girls, pregnant, breastfeeding women, and males receiving hormone replacement therapy for prostate cancer [6]. In 25%–40% of breast metastasis, a single mass is the initial presentation. It often presents as a painless, rapidly growing, superficial lesion in the upper outer quadrant, with no skin or nipple changes [9]. The left breast is more commonly involved (46.0%) than the right (40.3%) [1]. Our patient presented with a right breast lump, with involvement of multiple ipsilateral axillary lymph nodes. Axillary lymphadenopathy is an uncommon occurrence in primary lung cancer with an incidence of 0.75% [10]. Ultrasound findings in metastatic breast cancer include hypoechoic, oval, or round-shaped lesions with posterior acoustic enhancement [6]. Primary breast cancer, in contrast, commonly presents on ultrasound as masses with irregular borders and speculations [11]. On mammography, metastatic tumors appear as round masses with either well-circumscribed or irregular edges, without the speculations and calcifications that are frequently observed in primary breast cancer [6]. Histopathological examination, including tru-cut biopsy and IHC, is the key for definitive diagnosis. In this case, the biopsy of the breast mass showed poorly differentiated adenocarcinoma of lung origin. About 73%–88% of primary lung cancers and 2.4% of primary breast cancers are found to be TTF-1 positive, thus it is not reliable alone in ruling out a primary from metastatic breast cancer [12]. Hence, other immunostains like GATA3 are used, which is normally negative in lung adenocarcinoma and positive in <10% of the cases [13]. Napsin A and ROS1 could also be positive in ~90% and 2% of NSCLC cases, respectively, while they are typically negative in primary breast cancer [14, 15]. Management of metastatic breast cancer primarily focuses on treating the primary tumor. Unfortunately, cases of breast metastases typically have a bad prognosis, with >0% of the patients dying within a year [6].

## Conclusion

The diagnosis and management of breast metastasis from lung adenocarcinoma is challenging due to its rarity, especially in males. This case report underscores the importance of considering breast metastasis in the differential diagnosis of breast lesions in male patients, particularly those with a known primary malignancy like pulmonary adenocarcinoma. We encourage the reporting of similar cases to enhance understanding and improve the clinical approach to this unusual metastatic presentation.

## Data Availability

The data used to support the findings of this study are included in the article.

## References

[1] Koch A, Richter-Marot A, Wissler M, et al. Mammary metastasis of extramammary cancers: current knowledge and diagnostic difficulties. Gynecologie, Obstetrique, Fertilite 2013;41:653–9. 10.1016/j.gyobfe.2013.09.013. 24183577

[2] Ganti AK, Klein AB, Cotarla I, et al. Update of incidence, prevalence, survival, and initial treatment in patients with non–small cell lung cancer in the US. JAMA Oncol 2021;7:1824–32. 10.1001/jamaoncol.2021.4932. 34673888 PMC8532041

[3] Myers DJ, Wallen JM. Lung adenocarcinoma. In: Pi J (ed.), StatPearls [Internet]. St. Petersburg (FL): StatPearls Publishing; 2023.

[4] Stenbygaard LE, Sorensen JB, Olsen JE. Metastatic pattern in adenocarcinoma of the lung: an autopsy study from a cohort of 137 consecutive patients with complete resection. J Thorac Cardiovasc Surg 1995;110:1130–5. 10.1016/S0022-5223(05)80183-7. 7475142

[5] Lee AH . The histological diagnosis of metastases to the breast from extramammary malignancies. J Clin Pathol 2007;60:1333–41. 10.1136/jcp.2006.046078. 18042689 PMC2095576

[6] Lee SH, Park JM, Kook SH, et al. Metastatic tumors to the breast: mammographic and ultrasonographic findings. J Ultrasound Med 2000;19:257–62. 10.7863/jum.2000.19.4.257. 10759349

[7] Mirrielees JA, Kapur JH, Szalkucki LM, et al. Metastasis of primary lung carcinoma to the breast: a systematic review of the literature. Journal of Surgical Research 2014;188:419–31. 10.1016/j.jss.2014.01.024. 24560348

[8] Gargiulo P, Pensabene M, Milano M, et al. Long-term survival and BRCA status in male breast cancer: a retrospective single-center analysis. BMC Cancer 2016;16:1–11. 10.1186/s12885-016-2414-y. PMC4932666 27377827

[9] Ryu J, Lee S, Choi M, et al. 488 characteristics of metastasis in the breast from extramammary malignancies. EJC Supplements 2010;3:202. 10.1016/S1359-6349(10)70509-7. 20082359

[10] Satoh H, Ishikawa H, Kagohashi K, et al. Axillary lymph node metastasis in lung cancer. Med Oncol 2009;26:147–50. 10.1007/s12032-008-9097-4. 18821066

[11] Elsaeid YM, Elmetwally D, Eteba SM. Association between ultrasound findings, tumor type, grade, and biological markers in patients with breast cancer. Egypt J Radiol Nucl Med 2019;50:1–11. 10.1186/s43055-019-0048-1.

[12] Provenzano E, Byrne DJ, Russell PA, et al. Differential expression of immunohistochemical markers in primary lung and breast cancers enriched for triple-negative tumours. Histopathology 2016;68:367–77. 10.1111/his.12765. 26118394

[13] Laurent E, Begueret H, Bonhomme B, et al. SOX10, GATA3, GCDFP15, androgen receptor, and mammaglobin for the differential diagnosis between triple-negative breast cancer and TTF1-negative lung adenocarcinoma. Am J Surg Pathol 2019;43:293–302. 10.1097/PAS.0000000000001216. 30628926

[14] Turner BM, Cagle PT, Sainz IM, et al. Napsin A, a new marker for lung adenocarcinoma, is complementary and more sensitive and specific than thyroid transcription factor 1 in the differential diagnosis of primary pulmonary carcinoma: evaluation of 1674 cases by tissue microarray. Arch Pathol Lab Med 2012;136:163–71. 10.5858/arpa.2011-0320-OA. 22288963

[15] Yang J, Pyo J-S, Kang G. Clinicopathological significance and diagnostic approach of ROS1 rearrangement in non-small cell lung cancer: a meta-analysis: ROS1 in non-small cell lung cancer. Int J Biol Markers 2018;33:520–7. 10.1177/1724600818772194. 29874982

