# Correction to: From lung to breast: a rare case of metastatic lung adenocarcinoma presenting as a breast lump in a male patient

**DOI:** 10.1093/jscr/rjaf562

**Published:** 2025-09-25

This is a correction to: Elias Edward Lahham, Huda Maher Masri, Zeina Adnan Farhoud, Abrar Nidal Neiroukh, Mahmoud Ramahi, Farah Awad, Marwan Qubaja, From lung to breast: a rare case of metastatic lung adenocarcinoma presenting as a breast lump in a male patient, *Journal of Surgical Case Reports*, Volume 2025, Issue 3, March 2025, rjaf148, 10.1093/jscr/rjaf148

In the originally published version of this manuscript the following affiliation was missing for the author Marwan Qubaja: Faculty of Medicine, Al-Quds University, Main Campus, Abu Dis, PO Box 89, Palestine.

This error has been corrected.

